# Apical Periodontitis and Diabetes Mellitus Type 2: A Systematic Review and Meta-Analysis

**DOI:** 10.3390/jcm9020540

**Published:** 2020-02-17

**Authors:** Flor de Liz Pérez-Losada, Albert Estrugo-Devesa, Lissett Castellanos-Cosano, Juan José Segura-Egea, José López-López, Eugenio Velasco-Ortega

**Affiliations:** 1 Department of Odontostomatology, Faculty of Medicine and Health Sciences (School of Dentistry), University of Barcelona, L’Hospitalet de Llobregat, 08907 Barcelona, Spain; flordelizperezlosada@yahoo.es (F.d.L.P.-L.); albertestrugo@ub.edu (A.E.-D.); 2 Oral Health and Masticatory System Group (Bellvitge Biomedical Research Institute) IDIBELL, University of Barcelona, L’Hospitalet de Llobregat, 08907 Barcelona, Spain; 3 Department of Stomatology, School of Dentistry, University of Sevilla, 41009 Sevilla, Spain; lizettcastellanos@yahoo.es (L.C.-C.); segurajj@us.es (J.J.S.-E.); evelasco@us.es (E.V.-O.)

**Keywords:** apical periodontitis, diabetes mellitus, glycated haemoglobin, HbA1c, prevalence

## Abstract

Objective: Investigate if there is an association between apical periodontitis and diabetes mellitus. Material and methods: A bibliographic search was performed on Medline/PubMed, Scopus and Cochrane databases using the keywords apical periodontitis and diabetes mellitus. Published papers written in English and performed on animals or humans were included. Meta-analysis was performed using the OpenMeta (analyst) tool for the statistical analysis. The variables analyzed were the prevalence of Apical Periodontitis (AP) among teeth and patients with Diabetes Mellitus (DM). Results: Of the total studies found, only 21 met the inclusion criteria. Ten clinical studies on animals, ten studies on humans and a systematic review were included. Meta-analysis shows that the prevalence of teeth with apical periodontitis among patients with diabetes mellitus has an odds ratio of 1.166 corresponding to 507 teeth with AP + DM and 534 teeth with AP without DM. The prevalence of patients with AP and DM shows an odds ratio of 1.552 where 91 patients had AP + DM and 582 patients AP without DM. Conclusion: Scientific evidence suggests that there could be a common physiopathological factor between apical periodontitis and diabetes mellitus but more prospective studies are needed to investigate the association between these two diseases.

## 1. Introduction

The International Diabetes Federation states that in the year 2015, there was 415 million adults with diabetes in the world; this means that the number of people living with diabetes has quadrupled since 1980. Moreover, in the last decade, the prevalence of diabetes has increased more rapidly in low and medium income countries than in high income countries, which makes diabetes an important public health issue worldwide [1]. 

Diabetes mellitus is characterized by an inadequate carbohydrate, lipidic and protein metabolism, its primary aspect is hyperglycemia. This hyperglycemia acts as the main cause of incidence and progression of microvascular complication associated with the disease (retinopathy, nephropathy and neuropathy) [2]. In its ethiopathogenesis, influences a complex interaction of genetic and environment factors, establishing different causes of hyperglycemia. Among the factors associated to hyperglycemia are: differences in insulin secretion, lower glucose uptake or higher glucose production [3]. In the present time, it is known that the progressive defect in insulin production due to insulin resistance represents 90–95% of all individuals with diabetes mellitus type 2 (DM2) [4]. DM2 is considered a XXI century epidemic, not only for its magnitude but for its repercussions in cardiovascular disease, and is the main cause of death in developed societies [5]. Diabetes prevalence is high in countries such as Germany, Spain, Italy, France and United Kingdom, considering age an important risk factor. If we focus on Europe as an example, 37% of the population is over 50 years old and it is estimated that this number will increase to 44% by the year 2030, which means that a high increase of patients with diabetes is expected [5]. Glycated haemoglobin (HbA1c) levels test is considered the gold standard to control patients with diabetes (HbA1c) ≥6.5%. This test measures the average glycaemia of the last 2–3 months, allowing to assess the effectiveness of the treatment that the patient receives [4].

Amongst the environmental factors involved in the pathogenesis of type 2 diabetes, low-grade inflammation seems to occupy a prominent place [6]. This occurs when inflammatory stimuli of infectious origin, such as periodontal disease or apical periodontitis, both oral infections caused by Gram-negative bacteria, activate the innate immune system, causing a high level of pro-inflammatory interleukins [7]. Through this mechanism, apical periodontitis can induce or perpetuate an elevated chronic systemic inflammatory status, contributing to increased insulin resistance and poor glycemic control [8,9,10]. 

Apical periodontitis is the inflammatory response of the periapical tissue to Gram-negative bacterial infection of the dental pulp. Ninety percent of the cases is due to pulpal necrosis, secondary to tooth decay. This necrosis triggers an inflammatory and immune response when the polymicrobial and antigens products of the main or lateral root canals invade the periapical connective tissue [11]. 

Apical periodontitis is not only a local phenomenon, and for some time the medical and dental scientific community have analyzed the possible connection between apical periodontitis and systemic health. Endodontic medicine has developed, with increasing numbers of reports describing the association between periapical inflammation and systemic diseases [8,9]. Numerous studies in animals and humans suggest the existence of a link between apical periodontitis and some systemic diseases [8,9,12,13,14,15,16]. The possible connection between diabetes and apical periodontitis have been widely investigated in the last decade [8,17,18,19]. 

Analyzing what was previously described, we presume there is an association between diabetes and the prevalence of apical periodontitis, which makes the aim of this study to perform a systematic review and meta-analysis to investigate and examine if this association does exists.

## 2. Methodology

### 2.1. Focused Question

The present study followed the Preferred Reporting Items for Systematic Reviews and Meta-Analysis (PRISMA) guidelines [20]. Given the persistence of discrepancy between different studies, we propose to review the existing literature asking the following question: Is there any association between diabetes and the prevalence of apical periodontitis?

### 2.2. Search Strategy

The present study followed the Preferred Reporting Items for Systematic Reviews and Meta-Analysis (PRISMA) guidelines [20]. An electronic bibliographic search was performed in Medline/PubMed, Scopus and Cochrane databases, covering the period 2011–2019. The literature search was made using the following keywords apical periodontitis, diabetes mellitus, combined with the Boolean operator AND. After eliminating duplicates, the potential titles and abstracts were filtered based on the following criteria: Inclusion criteria were articles published in English or Spanish, performed in all animals or humans. Exclusion criteria were articles published in languages other than English. 

The criteria used for DM2 diagnosis on the investigations included, could be any of the following: (I) Fasting plasma glucose (FPG) ≥126 mg/dL (7.0 mmol/L). (II) Two-hour plasma glucose ≥200 mg/dL (11.1 mmol/L) during an oral glucose tolerance test (OGTT). (III) Occasional plasma glucose ≥200 mg/dL (11.1 mmol/L) (obtained at any time of the day regardless of the time spent since the last food intake) and severe classic hyperglycemic symptoms (polyuria, polydipsia, polyphagia) or hyperglycemic crisis [4].

### 2.3. Data Extraction and Analysis

To assess the methodological quality of the articles, the levels of evidence and degrees of recommendation were used according to the guidelines of the Oxford Centre for Evidence-Based Medicine (OCEBM) [21]. A single reviewer (FP), compiled all the information from the selected articles. Three reviewers (FP, LC and JSE), carried out the analysis of the articles; the articles in disagreement were discussed.

In order to analyze and synthesize the data, we extracted the following details from the studies: author and year of publication, study design, sample size, objective, results and results.

Pooled estimates from the studies were analyzed using a binary random-effects model meta-analysis. The variables analyzed were the prevalence of Apical Periodontitis (AP) among teeth and patients with Diabetes Mellitus (DM).

Forest plots were produced to graphically represent the odds ratio of AP in patients with DM, *p* = 0.05 was used as the level of significance. Heterogeneity was assessed with x^2^ test and I^2^ test. The OpenMeta (analyst) tool was employed in the statistical analysis.

## 3. Results

### 3.1. Study Selection

The bibliographic search adopting the search strategy yielded 27 articles in Medline/PubMed and Cochrane databases. Seventeen articles met the inclusion criteria and were included in the study, 8 studies were performed in animals [22,23,24,25,26,27,28,29], 8 studies in humans [30,31,32,33,34,35,36,37], and 1 systematic review/meta-analysis [11]. Ten articles were excluded because they did not meet the inclusion criteria.

**Figure 1:**
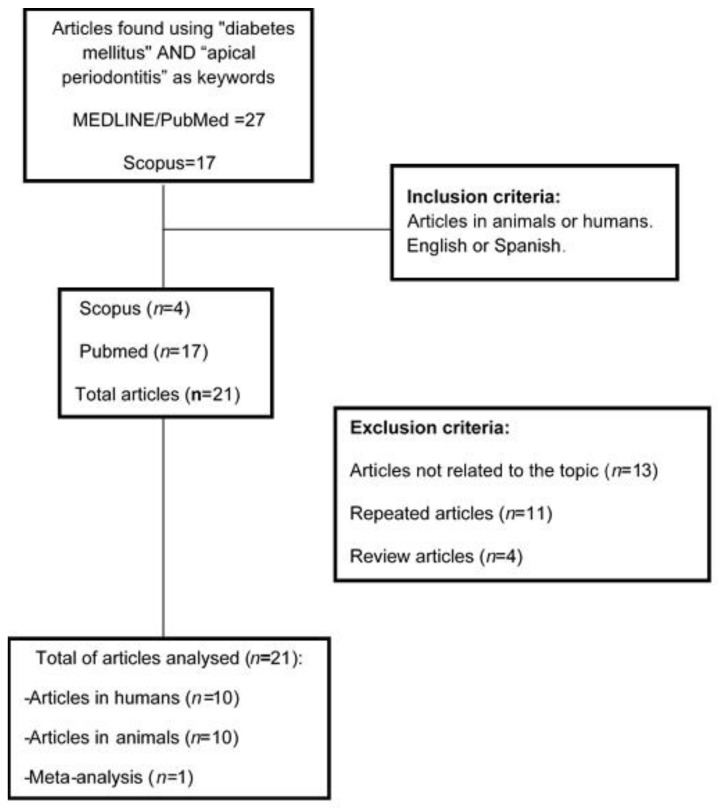
Flow diagram of selected articles.

The bibliographic search adopting the search strategy in the Scopus database yielded 17 articles. From the obtained research, 10 articles were duplicates, and 3 articles were excluded because they did not meet the inclusion criteria. Only four articles met the inclusion criteria: 2 studies in animals [38,39] and 2 studies in humans [40,41]. Finally, 21 article were included, 10 studies in animals [22,23,24,25,26,27,28,29,38,39], 10 studies in humans [30,31,32,33,34,35,36,37,40,41] and 1 systematic review [11] (Figure 1).

### 3.2. Quality Assessment

Levels of evidence in animals. Analysis of the levels of evidence in animal studies showed that nine studies had a B recommendation (90%), with a level of evidence of 3b [22,38], 7 studies had a B recommendation with a level of evidence 2b [24,25,26,27,28,29,39] and one study with grade of recommendation A and level of evidence 1b (10%) [23].

Of the animal studies, 100% were performed in mice, representing 595 mice, in five studies the n was greater than 80, representing 408 mice [24,25,26,29,38]. In four studies, the n was greater than 40 with a total of 167 mice [22,27,28,39] and in a single trial the n was of 20 mice [23].

Levels of evidence in humans. An association between apical periodontitis and diabetes was found in seven of these experimental studies with an *n* value of 455 positive cases (76.47%) [11,31,33,40,41]. However, in one study [32] with an *n* value of 62 individuals (4.28%), they reported no statistical significance between these two entities and on two studies, they did not report whether or not an association was established, main outcomes are reflected on Table 1 and Table 2.

Analysis of the levels of evidence in human studies showed 10 articles, classified as the following: 6 cross-sectional studies descriptive type, representing 60% of the articles included [31,33,35,37,40,41]; three prospective studies 30% [30,34,36] and 1 retrospective study 10% [32]. In these studies, 2088 patients were analyzed, in which the n value was highly variable: 1214 patients [30], 291 patients [35], 90 patients [31], 62 patients [32], 83 patients [33], 46 patients [36], 100 patients [40], 80 patients [34], and 122 patients [41].

The relationship between diabetes mellitus and apical periodontitis is clearly established in four studies [31,33,40,41] with an n value of 395 corresponding to 22.55% of the studied population, 285 patients had diabetes mellitus type 2 representing 72.15% of the sample studied in this four investigations. In all of them, the Periodontitis Apical Index (PAI) was used to assess AP, except in the studies by Marotta et al. 2012 [31] and Rudranaik et al. 2016 [34] that used Strindberg criteria. In contrast, the study by Ferreira et al. [32] with an n of 62 did not found a significant difference between these two entities (Table 1 and Table 2).

**Figure 2:**
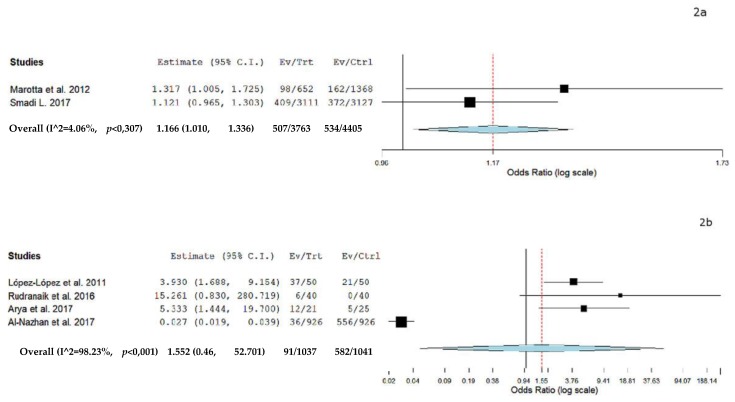
(**a**) Forest plot of prevalence of teeth with Diabetes Mellitus (DM) and Apical Periodontitis (AP); (**b**) Forest plot of prevalence of patients with DM and AP. Overall (I^2 = 98.23%, *p* < 0.001), 1.552 (0,46, 52.701), 91/1037, 582/1041.

A separate meta-analysis was performed to analyze the odds ratio of AP and DM. Six case control studies were included in the meta-analysis (two which assessed the prevalence of teeth with AP among patients with DM and control and four assessing prevalence of patients with AP and DM). Figure 2a shows the results of the meta-analysis from the data extracted from the studies [31,35] assessing the prevalence of teeth with AP among patients with DM, indicating an odds ratio of 1.166 and a *p* value = 0.02 (95% CI: 1.018 to 1.336, heterogeneity I^2^ = 4.06%, *p* = 0.30). Figure 2b demonstrates the results of the meta-analysis from the data extracted from the studies [34,36,37,40] assessing the prevalence of patients with AP and DM indicating an odds ratio of 1.552 and a *p* value = 0.807 (95% CI: 0.046 to 52.701, heterogeneity I^2^ = 98.23%, *p* = < 0.001).

## 4. Discussion

There is evidence in animal and human studies showing an association between apical periodontitis and diabetes mellitus [22,23,24,25,26,30,31,32,33,40,41], although the type of study, sample size and level of scientific evidence are different in each one of them (Table 1 and Table 2).

### 4.1. Animal Studies

Several experimental studies have found an effect of diabetes on the development of carious lesions and apical periodontitis. The incidence and severity of carious lesions, alveolar resorption and periapical lesions are higher in rats with chronic diabetes [22] (Oxford level 3b/B), [39] (Oxford level 2b/B), aggravating dental caries [38] (Oxford level 3b/B). These findings suggest that apical periodontitis in diabetic animals is a secondary consequence of dental caries.

Experimental studies support the influence of apical periodontitis on glucose and glycated haemoglobin levels of diabetic animals. Studies carried out using mice as an experimental model, have reported higher glycaemia, and greater periapical inflammatory infiltrate with more bone resorption in diabetic mice with apical periodontitis, compared to control animals [24] (Oxford level 2b/B). Additionally, diabetic mice with periapical lesions presented higher levels of Hb1Ac, suggesting that concomitant periapical infections may impair metabolic control of diabetes.

Vice versa, experimental studies in diabetic animals support the potentiating effect of diabetes on periapical inflammation. Diabetic animals showed greater radiolucent periapical lesions compared to controls [25], suggesting that diabetes increases periapical lesions. Moreover, diabetes mellitus accelerated the development and progression of AP, producing an increase in the erythrocyte cell media, as well as leukocytes and neutrophil counts [26] (Oxford level 2b/B). Apical periodontitis significantly increases the levels of inflammatory interleukins in diabetic animals with apical periodontitis. Diabetic animals show a significant increase of IL-17 levels when compared to control mice [25]. Increased level of IL-17 was also found in diabetic rats without AP [27] (Oxford level 2b/B), suggesting that diabetes plays an important role in the increment of IL-17. These findings, together with those of Prieto et al. 2017 [28] (Oxford level 2b/B), who found more aggressive inflammatory infiltrate in the periapical area of diabetic animals, with lower levels of serum albumin and increased level of antioxidant uric acid, support the link between diabetes and periapical inflammation. A recent study has reported a more intense periapical inflammatory infiltrate, with larger sizes of bone reabsorption, in diabetic animals [42].

### 4.2. Human Studies

Regarding the level of evidence and degree of recommendation (OCEBM), a type B recommendation was found in all studies [30,31,32,33,34,35,36,37,40,41] and a level of evidence of 2b [30,34,36] and 3b in the rest of the investigations [31,32,33,35,37,40,41].

Several epidemiological studies carried out in humans reported findings suggesting that diabetes is associated to periapical lesions. A cross-sectional study [40] (Oxford level 3b/B) found higher prevalence of apical periodontitis in patients with diabetes mellitus (OR = 3.9; *p* = 0.002), as well as higher number of Randomized Clinical Trials –RCT- (OR = 2.3; *p* = 0.043). Another cross-sectional study [31] [Oxford level 3b/B] also found higher prevalence of apical periodontitis in teeth of diabetic patients (15%), compared to controls (12%) (*p* = 0.05). Similar results have been reported in other cross-sectional studies [32,35,37], but the differences were not significant. However, the results of these epidemiological studies, most cross-sectional, cannot be interpreted as a proof of causal relationship. Cross-sectional studies only provide evidence of association [17].

Some longitudinal studies also find an association between apical periodontitis and the outcome of RCT. NG YL et al. 2011 [30] (Oxford level 2b/B) performed a prospective clinical trial analyzing the factors influencing tooth survival following primary or secondary root canal treatment. They concluded that the survival of teeth with RCT, either primary or secondary, after 4 years of follow-up was of 95%, being diabetes one of the significant patient factors. Other prospective studies have found that periapical lesions in diabetic healed slowly, compared to control subjects [36]. Moreover, it has been reported that patients with poor controlled diabetes (15%) did not show successful healing of the periapical lesions at 1 year follow up [34] (Oxford level 2b/B). Additionally, it has been reported that the frequency of periapical lesions in patients with long-term diabetes was higher than in patients with short-term diabetes [41] (Oxford level 3b/B).

The association between the diabetic status and a slower healing of the periapical lesion has been confirmed by two systematic reviews with meta-analysis [11,43]. These systematic reviews conclude that diabetes is a main pre-operatory risk factor for RCT. Moreover, an umbrella review recently published on the association between diabetes and the outcome of RCT conclude that the prognosis of RCT is worse in diabetic patients [44]. The biological mechanisms by which diabetes mellitus can influence the healing of periapical tissues, affecting the outcome of RCT, are mainly three: impaired innate immunity, hyperglycaemia and the formation of irreversibly glycated-proteins forming advanced glycation end products (AGEs) [8].

Finally, several studies have analyzed the relationship between apical periodontitis and the metabolic control of diabetes. Sánchez-Domínguez et al. 2015 [33] (Oxford level 3b/B) performed a cross-sectional study evaluating the glycated hemoglobin levels of diabetic patients in relation with their periapical status. Good controlled diabetes was considered when HbA1c <6.5% and poor controlled diabetes when HbA1c ≥6.5%. Their results revealed that the periapical state was significantly associated with HbA1c levels. Multivariate logistic regression analysis showed that worse periapical status correlated significantly with HbA1c levels ≥6.5% in patients with type 2 diabetes (*p* = 0.03). On the other hand, higher HbA1c levels have been associated to higher prevalence of RFT and RFT with AP [35]. The biological mechanism by which apical periodontitis could alter the metabolic control of diabetes, increasing HbA1c levels, would be related to the induction of a systemic inflammatory status, contributing to increased insulin resistance and poor glycaemic control [8,17].

## 5. Conclusions

The analysis of the studies included in this review suggests that there is an association between apical periodontitis and diabetes mellitus. Animal studies support a causal link between diabetes and the size and healing of periapical tissues. However, the quality of the epidemiological studies carried out in humans is medium or low. More prospective studies in humans are needed to investigate the association between these two diseases.

## Figures and Tables

**Table 1:** Main features of animal studies included on the review.

Author, Year	Level of Evidence/Degree of Recommendation	Sample and Groups	Association AP and DM	Results
Kodama et al., 2011 [39]	2b/B	F344 Mice, *n* = 40 DM Mice: ♂ (*n* = 10); ♀ (*n* = 10) Non-DM Mice: ♂ (*n* = 10); ♀ (*n* = 10)	Yes	The incidence and severity of caries, alveolar bone resorption and periapical lesions were higher in rats with chronic DM.
Sano et al., 2011 [38]	3b/B	*n* = 88 Mice Mice ♂: *n* = 68 (DM and no-DM) Mice ♀: *n* = 20 (DB and no-DM)	Yes	Diabetes increase dental caries and suggest that apical periodontitis is secondary to dental caries in non-DM mice.
Nakahara et al., 2012 [22]	3b/B	♀ F344 Mice, *n* = 47 TG: *n* = 30 *(1 dose of Aloxane, 35 mg/kg corporal weight)* CG: *n* = 17 untreated	Yes	Mice treated with Aloxane developed more carious lesions and progressive periodontitis.
Wolle et al., 2013 [23]	1b/A	♂ Wistar Mice, *n* = 20 TG: *n* = 15; mice with DM *(receive 20% D-Glucosein drinking water, 10 mL/kg/9 weeks)* GC: *n* = 5; no-DM mice *(receive filtered/9 weeks)*	No	No significant differences were found between groups. Tempol does not improve the outcome of injuries associated with endodontic teeth.
Cintra, et al., 2014 [24]	2b/B	Albinos Wistar Mice, *n* = 80 (10 each group) ^#^ G1: GC; G2:AP; G3: PD; G4: AP + PD; G5: DM, G6: DM + AP; G7:DM + PD; G8: DM + AP + PD	Yes	Mice with oral pathology (AP + PD) had a significant increase in IL-17 compared to mice without oral pathology.
Cintra, et al., 2014 [25]	2b/B	Albinos Wistar Mice, *n* = 80 (10 each group) ^#^ G1: GC; G2:AP; G3: PD; G4: AP + PD; G5: DM, G6: DM + AP; G7:DM + PD; G8: DM + AP + PD	Yes	Oral infections affect blood sugar levels in DM mice and increase HbA1c levels in DM and normoglycemic mice.
Cintra, et al., 2014 [26]	2b/B	Albinos Wistar Mice, *n* = 80 (10 each group) ^#^ G1: GC; G2:AP; G3: PD; G4: AP + PD; G5: DM, G6: DM + AP; G7:DM + PD; G8: DM + AP + PD	Yes	DM increases the development and progression of AP and PD, causing an increase in the cellular mean of erythrocytes, leukocytes and neutrophils. Both oral infections increased the total number of leukocytes, neutrophils, lymphocytes and glucose concentrations in mice with DM.
Azuma et al., 2017 [27]	2b/B	♀ Winstar mice *n* = 40 (10 each group) Normoglycemic rats (N); Normoglycemic rats with apical periodontitis (N-AP); rats with experimental diabetes (ED), and rats with experimental diabetes and apical periodontitis (ED-AP)	Nr	AP did not impact the levels of IL-17 in hepatic and renal tissues, irrespective of the presence or absence of diabetes. There is an increase of IL-17 levels in the periapical region of diabetic rats without AP (ED) as compared to control rats (N), and an increase of IL-17 levels in AP rats with experimental diabetes (ED-AP) was observed as compared to the N-AP.
Prieto et al., 2017 [28]	2b/B	♀ Winstar mice *n* = 40 (10 each group) G1:CG, G2: AP, G3: DM, G4: DM + AP	Yes	Microscopically in the groups with AP (AP and DM + AP), an increase in the intensity and extent of the inflammatory infiltrate was noted, periapical lesions in the diabetic rats were higher and more aggressive compared with that in normoglycemic rats, AP associated with diabetes reduced the serum levels of albumin and increased the endogenous antioxidant uric acid.
Ferreira et al., 2017 [29]	2b/B	♀ Winstar mice *n* = 80 (10 each group) G1:CG, G2: AP, G3:PD, G4:AP + PD, G5:DM, G6: DM + AP, G7: DM + PD, G8:DM + AP + PD	Nr	The presence of oral infections increased blood glucose concentrations in diabetic rats. DM + PD and DM + AP + PD groups had higher mean values of platelet count with statistical difference compared to CG and AP groups.

AP: apical periodontitis; CG: control group; DM: diabetes mellitus; DM2: diabetes mellitus type 2; M: meta-analysis; no-DM: no diabetes mellitus; HbA1c: glycated haemoglobin; *n*: sample; PD: periodontal disease; TG: treatment group; #: Diabetes mellitus was induced with Streptozotocin, apical periodontitis with oral exposition and periodontal disease by periodontal ligature. Nr: not reported.

**Table 2:** Main features of human studies included on the review.

Author, Year	Level of Evidence/Degree of Recommendation	Sample and Groups	Association AP and DM	Results
López-López et al., 2011 [40]	3b/B/CS	*n* = 100 TG: *n* = 50, patients with DM2 CG: *n* = 50, patients without DM	Yes	DM2 is significantly associated with a higher prevalence of AP and root canal treatment.
NG YL et al., 2011 [30]	2b/B/ PCT	*n* = 1617 teeth in 1214 patients. Group 1: Primary RCT Group 2: Secondary RCT	Nr	A 95% survival of primary and secondary root canal treatment after 4 years was found with 13 common factors, DM being one of the significant patient’s factors.
Marota et al., 2012 [31]	3b/B/CS	*n* = 90 TG: *n* = 30, patients with DM2 CG: *n* = 60, patients without DM	Yes	A higher prevalence was found in patients with DM than in patients without previous history of DM.
Ferreira et al., 2014 [32]	3b/B/RCT	*n* = 62 TG: *n* = 37 teeth CG: *n* = 25 teeth	No	No significant difference was found between both groups for AP.
Mesgarani et al., 2014 [41]	3b/B/CS	*n* = 122 Long-term DM patients (>48 months): *n* = 85 Short-term DM patients (<48 months): *n* = 37	Yes	The frequency of AP was more significant in patients with long-term DM than in those with short-term DM.
Sánchez-Dominguez et al., 2015 [33]	3b/B/CS	*n* = 83 CG: *n* = 24, good control DM (HbA1c < 6.5%) TG: *n* = 59, poor control DM (HbA1c > 6.5%)	Yes	AP is significantly associated with HbA1c levels in patients with DM and root canal treatment.
Rudranaik et al., 2016 [34]	2b/B/PCT	*n* = 80 Group 1: *n* = 40, patients without DM Group 2: *n* = 40, patients with DM2	Nr	Patients with diabetes were more prone for chronic periapical disease with larger lesions. Healing outcome at one year was unsuccessful in poor controlled patients with diabetes when compared to fair and good controlled patients in group 2.
Segura-Egea et al., 2016 [11]	1a/A/M	*n* = 7 Epidemiological studies: 1593 teeth with root canal treatment GT: *n* = 582 patients with DM GC: *n* = 1011 patients without DM	Yes	AP is significantly associated with diabetes with a higher prevalence of periapical lesions on the teeth with root canal treatment.
Smadi L. 2017 [35]	3b/B/CS	*n* = 291 patients Group 1: *n* = 145 patients; *n* = 3111; 409 teeth with DM + AP Group 2: *n* = 146 patients; *n* = 3127; 372 teeth with No DM + AP	Nr	A higher prevalence of AP in DM patients but this difference was not statistically significant.
Arya et al., 2017 [36]	2b/B/PCT	*n* = 46 Group 1: 21 patients with DM/12 with DM + AP Group 2: 25 patients without DM/5 No DM + AP	Nr	Periapical healing showed a significantly lower success rate at 1 year follow up in the type 2 diabetic group than the nondiabetic group. However, even among type 2 diabetics, 90% of cases showed an improved periapical status. No significant difference in healing between good controlled and poor controlled patients or those with raised HbA1c levels was found.
Al-Nazhan et al., 2017 [37]	3b/B/CS	*n* = 926 patients 36 patients with DM + AP 556 with no DM + AP	Nr	This study revealed a higher prevalence of AP in diabetic subjects, although the sample of diabetic patients was small.

AP: apical periodontitis; CG: control group; CS: cross-sectional study; DM: diabetes mellitus; DM2: diabetes mellitus type 2; M: meta-analysis; no-DM: no diabetes mellitus; HbA1c: glycated haemoglobin; *n*: sample; PD: periodontal disease; PCT: prospective clinical trial; RCT: retrospective clinical trial; TG: treatment group; Nr: not reported.
